# Characterization of yeast mutant strains for starter culture in Arabica coffee fermentation

**DOI:** 10.1038/s41598-024-56298-6

**Published:** 2024-03-13

**Authors:** Yaowapa Meeampun, Titiporn Panyachanakul, Siritron Samosorn, Kulvadee Dolsophon, Rossaporn Jiamjariyatam, Wanlapa Lorliam, Jantima Arnthong, Surisa Suwannarangsee, Prapakorn Tantayotai, Sukhumaporn Krajangsang

**Affiliations:** 1 Department of Microbiology, Faculty of Science, Srinakharinwirot University, 114 Sukhumvit 23, Wattana, 10110 Bangkok Thailand; 2 Department of Chemistry, Faculty of Science, Srinakharinwirot University, 114 Sukhumvit 23, Wattana, 10110 Bangkok Thailand; 3 National Center for Genetic Engineering and Biotechnology (BIOTEC), National Science and Technology Development Agency (NSTDA), 113 Thailand Science Park, Klong Luang, 12120 Pathumthani Thailand; 4 Department of Science and Bioinnovation, Faculty of Liberal Arts and Science, Kasetsart University Kamphaeng Saen Campus, Nakhon Pathom, 73140 Thailand

**Keywords:** Arabica coffee, Fermentation, Mutation, Yeast, Starter culture, Biotechnology, Microbiology

## Abstract

Arabica coffee is the most popular and best-selling type of coffee. During coffee fermentation, microorganisms are essential for the production of metabolites and volatile compounds that affect coffee flavor quality. This work aimed to study the mutation, selection, and characterization of the *Wickerhamomyces anomalus* strain YWP1-3 as a starter culture to enhance the flavor quality of Arabica coffee. The results revealed that six mutants could produce relatively high levels of the pectinase enzyme on pectin agar media and exhibited high activity levels, ranging from 332.35 to 415.88 U/ml in mucilage broth. Strains UV22-2, UV22-3, UV41-1 and UV32-1 displayed higher levels of amylase activity than did the wild type. The UV22-2 and UV22-3 mutants exhibited the highest pectin degradation indices of 49.22% and 45.97%, respectively, and displayed significantly enhanced growth rates in nitrogen yeast base media supplemented with various sugars; thus, these mutants were evaluated for their ability to serve as a starter for fermentation of Arabica coffee. The cupping scores of coffees derived from UV22-2 and UV22-3 were 83.5 ± 1.5 and **82.**0 ± 2.14, respectively. The volatile compounds in the roasted coffee fermented by UV22-2 were analyzed by GC‒MS, which revealed higher levels of furfuryl alcohol and furfuryl acetate than did the other samples. These findings suggested that UV22-2 could be an influential starter culture for Arabica coffee fermentation.

## Introduction

Coffee, a widely consumed nonalcoholic beverage, is highly regarded worldwide. Among the primary coffee varieties, Arabica stands out due to its superior sensory properties, characterized by a rich aroma and flavor with relatively few unpleasant notes, making it highly sought after in the market^1^. Coffee quality is influenced by factors such as cultivar, growing region, and postharvest process^2^. The production of coffee typically involves three methods: the wet process, dry process, and semidry process^3^. Arabica coffee is predominantly subjected to a wet process, which involves depulping ripe fruits, submerging fermentation, and drying until a final moisture content of 10–12% is reached^4,5^. Currently, this process relies on naturally occurring microorganisms present in the raw materials, leading to unregulated and variable coffee quality.

Microorganisms play a crucial role in the degradation of the coffee pulp and mucilage layer, producing acids and other metabolic compounds that can permeate through the parchment layer to the coffee beans in wet processes^3^. The mucilage layer of depulped coffee beans consists of water, protein, sugar, pectic materials, and ash^6,7^. Notably, the breakdown of macromolecules, including carbohydrates, proteins, and polyphenols, by extracellular enzymes and organic acids generated during wet fermentation leads to the production of aroma precursors such as reducing sugars, amino acids, and chlorogenic acids. Secondary metabolites generated during fermentation potentially contribute to the unique flavors of coffee^7^. During the process of coffee fermentation, a variety of metabolites are generated by microorganisms. The level of microbial activity and the extent of fermentation play crucial roles in shaping the concentrations of free sugars (such as glucose and fructose) and free amino acids that persist around the coffee bean. These components, in turn, contribute to the formation of Maillard compounds and volatiles during the subsequent roasting process^8^.

Controlled fermentation using selected microbes as starter cultures has emerged as a means to modulate coffee flavor. Studies have shown that employing starter cultures, such as *Pichia fermentans* YC5.2, in controlled coffee bean fermentations during wet processing supplemented with sucrose can yield high-quality coffees with distinct characteristics, such as an intense “vanilla” taste and “floral” aromas^2,8^. Various pectinolytic yeasts, including *Wickerhamomyces anomalus* KNU18Y3, *Saccharomycopsis fibuligera* KNU18Y4, *Papiliotrema flavescens* KNU18Y5 and KNU18Y6, *Pichia kudriavzevii* KNU18Y7 and KNU18Y8, and *Saccharomyces cerevisiae* KNU18Y12 and KNU18Y13, have been isolated from coffee fermentation during wet processing for their potential as starter cultures^9^. In a previous report, *Wickerhamomyces anomalus* YWP1-3 was isolated from an Arabica coffee farm located in Thailand and demonstrated potential for pectinase production and growth in various culture media^10^. Research on coffee fermented with *Wickerhamomyces anomalus* YWP1-3 (condition ss02) has revealed unique cupping notes, including pepper, nutty, spicy, perfume, rose, floral, caramel, bell pepper, long beans, roast, orange, and green apple notes^11^. Therefore, the quality of coffee is improved when specific yeasts are employed as starters, and the synthesis of pectinase can be used to screen for desired strains.

Mutagenesis is a process that can create new genotypes through either spontaneous mutations or induced mutations. Induced mutations occur when genetic material is exposed to physical or chemical agents known as mutagens. Commonly used conventional mutagens for strain improvement include N-methyl-N'-nitro-N-nitrosoguanidine (NTG), ethyl methanesulfonate (EMS), hydroxylamine (NH_2_OH), nitrous acid (HNO_2_), and ultraviolet (UV) radiation. These mutagens can induce changes in genetic material, leading to the generation of novel genotypes with potentially beneficial traits^12–14^. In the context of pectinase production, UV mutagenesis has been employed to increase the production of pectinases in *Aspergillus niger* C28B25 through solid-state fermentation (SSF) of coffee pulp^15^. Additionally, UV and NTG have been utilized to improve pectinase production in *Aspergillus sojae*^14^. However, to date, there have been no reports on the utilization of mutant yeast as a starter culture for coffee fermentation.

The aims of this study were to generate *W. anomalus* YWP1-3 mutants using a chemical mutagen, EMS, and a physical mutagen, UV, and to assess the potential of these mutant strains for use as starter cultures in coffee fermentation.

## Materials and methods

### Yeast strain and Arabica coffee used in this study

The wild-type strain *Wickerhamomyces anomalus* YWP1-3 was originally obtained from coffee production processes and previously evaluated as a prospective starter culture for wet coffee fermentation^10^. The strain was previously identified by analyzing the gene sequence similarities of the small-subunit (SSU) rRNA. For the DNA sequence analyses, the 18S rRNA gene was amplified by using the primers NS1 (5′-GTAGT CATAT GCTTG TCTC-3′) and NS24 (5′- AAACCTTGT TACGACTTTTA-3′). The stock culture was preserved at − 20 °C and subsequently revived using YM broth, which comprised 3 g/L yeast extract, 3 g/L malt extract, 5 g/L peptone, and 10 g/L glucose and was maintained at a pH of 6.0. Arabica coffee (*Coffea Arabica* L.) cherries were obtained from coffee farms belonging to the Community Enterprise Groups in Thep Sadet Subdistrict, Doi Saket District, Chiang Mai Province, Thailand. The plant collection and use procedures were in accordance with all the relevant guidelines.

### UV and EMS resistance of *W. anomalus* YWP1-3

The starter culture for the mutation experiment was prepared as follows. The wild-type strain was cultured in a tube containing 5 ml of YM broth and incubated at 30 °C with shaking at 150 rpm for 24 h. This starter culture was then transferred to a flask containing 200 ml of YM broth at a final concentration of 10% (v/v). The flask was incubated at 30 °C for 6 h. After incubation, the culture broth was centrifuged at 8000 rpm for 10 min at 4 °C. The resulting cell pellet was washed twice with 30 ml of 0.85% NaCl, resuspended in 0.85% NaCl and adjusted to achieve an optical density of 1 at a wavelength of 600 nm (2 × 10^8^ colony-forming units (CFU) per milliliter).

For UV radiation, a 10 ml cell suspension was transferred into a sterilized petri dish with a 1-inch stent and placed on a stirrer inside a sterilized UV box. The yeast cells were exposed to UV light (UV radiation intensity of 51 μW/cm^2^) for various durations (0, 30, 60, 90, and 120 s). The plated cells were then incubated in a dark environment (to deactivate photoreactivation) for a period of 48 h to allow for growth.

For ethyl methanesulfonate (EMS) induction, the wild-type strain was prepared, and its concentration was adjusted to 2 × 10^8^ CFU/ml. EMS at a concentration of 20 g/l was added to 1 ml of the cell suspension for different time intervals (10, 20, 30, 40, 50, 60, and 90 min). To prevent mutagenesis caused by EMS, 1 ml of 5% sodium thiosulfate was added. Subsequently, the cell suspension was centrifuged at 8000 rpm for 10 min at 4 °C, and the resulting pellet was resuspended in sterilized water.

The quantification of viable cells following the mutagenesis treatments was conducted by plating 0.1 ml of the treated cell suspension onto pectin agar (composed of 10 g/L pectin, 1 g/L NaNO_3_, 1 g/L KCl, 1 g/L K_2_HPO_4_, 0.5 g/L MgSO_4_, 0.5 g/L yeast extract, and 20 g/L agar adjusted to pH 7.0) immediately after UV irradiation and EMS treatment. After the incubation period, the resulting colonies were counted to determine the number of viable cells.

The screening and selection of mutant yeast strains were performed using the replica plating technique and a modified version of Gram's iodine assay. In this assay, Gram's iodine solution was added to pectin agar plates, followed by incubation for 5 min. Subsequently, the plates were washed with distilled water. Mutant strains exhibiting a clear zone size larger than that of the wild type were selected because they indicated potential phenotypic changes. These selected mutant strains were then chosen for further study and analysis.

### Characterization of *W. anomalus* YWP1-3 mutant strains

Wild-type and selected mutant strains of *W. anomalus* YWP1-3 were characterized to assess their suitability for coffee fermentation. The characterization included examining colony morphology and yeast cell morphology on YMA agar after 24 h of growth using light microscopy. The growth ability of the strains in various media, namely, YM broth, pectin broth, and mucilage broth, was evaluated by measuring the optical density at 600 nm over a 72 h period^10^. Pectinase activity was determined using the pectin degradation index (PDI), which was obtained from cultures grown on pectin agar and incubated at 30 °C for 7 days. Crude enzymes extracted from the mucilage broth of the yeasts were used to measure pectinase, cellulase, and amylase activities via the DNS method. Pectinase, cellulase and amylase activities were assayed by using 1% (w/v) pectin, 0.5% (w/v) carboxymethylcellulose and 0.5% soluble starch in 0.1 M sodium phosphate buffer (pH 7.0) as substrates, respectively^16^. All reaction mixtures were incubated at 50 °C for 30 min. The reaction was terminated by adding DNS reagent, boiling for 5 min, and measuring the absorbance at 540 nm. One unit of enzyme activity was defined as the amount of enzyme that liberated 1 μmol of reducing sugar per min under the described conditions.

Additionally, the ability of the wild-type and selected mutant strains to assimilate various carbohydrate compounds (maltose, fructose, mannose, arabinose, lactose, rhamnose, glucose, xylose, galactose, and sucrose) commonly found in coffee cherries was determined using nitrogen base broth according to previous reports^10^.

### Arabica coffee fermentation

#### Yeast strains

*Wickerhamomyces anomalus* YWP1-3 (wild type), mutant UV22-2, and mutant UV22-3 were used as starter strains in this study. The stock culture was stored at − 20 °C and was reactivated in YM broth.

#### Starter culture preparation

Fresh cultures of each strain were initiated by transferring them into 50 ml of YM broth and then incubating at room temperature for 24 h while agitating at 150 rpm. Subsequently, 5 ml of the culture broth was transferred to 50 ml of YM broth and incubated at room temperature for 48 h with continuous shaking at 150 rpm. Subsequently, the culture was centrifuged at 10,000 rpm for 10 min to collect the cell pellet of all strains, which was then utilized as the starter culture for the coffee fermentation process.

#### Arabica coffee fermentation

The starter cultures of each strain were blended (10% inoculum) with a mixture of 10 L of water and 7 kg of freshly cleaned and depulped Arabica coffee beans within a tank. These depulped coffee beans were incubated for 48 h at ambient temperatures ranging from 13 to 25 °C. This process took place at the Community Enterprise Groups in Thep Sadet Subdistrict, Doi Saket District, Chiang Mai Province, Thailand. Following the 48 h fermentation period, the coffee beans were thoroughly washed. A control fermentation batch was also prepared using the same method but without the addition of the starter culture.

#### Sensory analysis

Green coffee beans underwent roasting at a temperature of 115.6 °C until they reached a target Agtron gourmet color scale rating of 55–65 Agtron. Subsequently, sensory testing using SCAA Protocols^17^ was conducted within 24 h of the roasting process. The ideal proportion is 8.25 g of coffee per 150 ml of water. The water was freshly drawn and heated to approximately 93 °C before being poured onto the ground coffee. The hot water was poured directly onto the measured grounds until the cup was filled the rim, ensuring that all the ground was thoroughly wet. The ground was allowed to thaw undisturbed for 3–5 min before being allowed to brew. The certified 3 Q-Arabica graders assessed the quality of coffee flavor by evaluating attributes such as fragrance/aroma, flavor, aftertaste, acidity, body, balance, uniformity, clean cup, sweetness, defects, and overall impression.

#### Evaluation of volatile compounds in roasted coffee by gas chromatography–mass spectrometry (GC–MS)

The roasted coffee samples were transformed into a powder using a CryoMill (Retsch, Germany) with liquid nitrogen. One gram of roasted coffee powder was precisely measured and placed in a 20 ml headspace vial. The vials were then sealed and preheated for 10 min at 80 °C. Subsequently, volatile compounds were extracted by a solid-phase microextraction (SPME) fiber (50/30 µm DVB/CAR/PDMS, SUPELCO, PA) for 20 min. A GC injector port was used to desorb the fiber at a temperature of 250 °C for 5 min. The separation of volatile compounds in roasted coffee was carried out using gas chromatography–mass spectrometry (Agilent 7890A GC-7000 Mass Triple Quad), which was set with a capillary column (DB-WAX, 60 m × 0.25 mm × 0.25 μm, J&W Scientific, Folsom, CA) and a quadrupole mass detector (Lee et al., 2015). Helium gas was used as the carrier gas with a consistent flow rate of 0.8 mL/min and a split ratio of 5:1. The temperature of the GC oven was set at 32 °C for 10 min, increased to 40 °C at a rate of 3 °C/min, held for 15 min and increased to 230 °C at 4 °C. The mass spectrometer was operated in electron ionization mode according to the methods of Mahingsapun et al.^11^. The identification of volatile compounds was achieved by comparing the mass spectra with the NIST mass spectral libraries (National Institute of Standards, 2011 version). The mass spectra were qualitatively compared with the Wiley 10n 14.l mass spectral database using a match factor greater than 80%.

### Statistical analysis

Statistical analyses were performed using IBM SPSS Statistics software (version 23.0, IBM Corp., Armonk, N.Y., USA). Analysis of variance (ANOVA) was conducted to determine significant differences among the groups, followed by post hoc comparisons of means using the LSD. The level of significance was set at a two-sided *p* value of less than 0.05.

## Results

### Mutation and screening of *W. anomalus* YWP1-3 mutant strains

**Figure 1:**
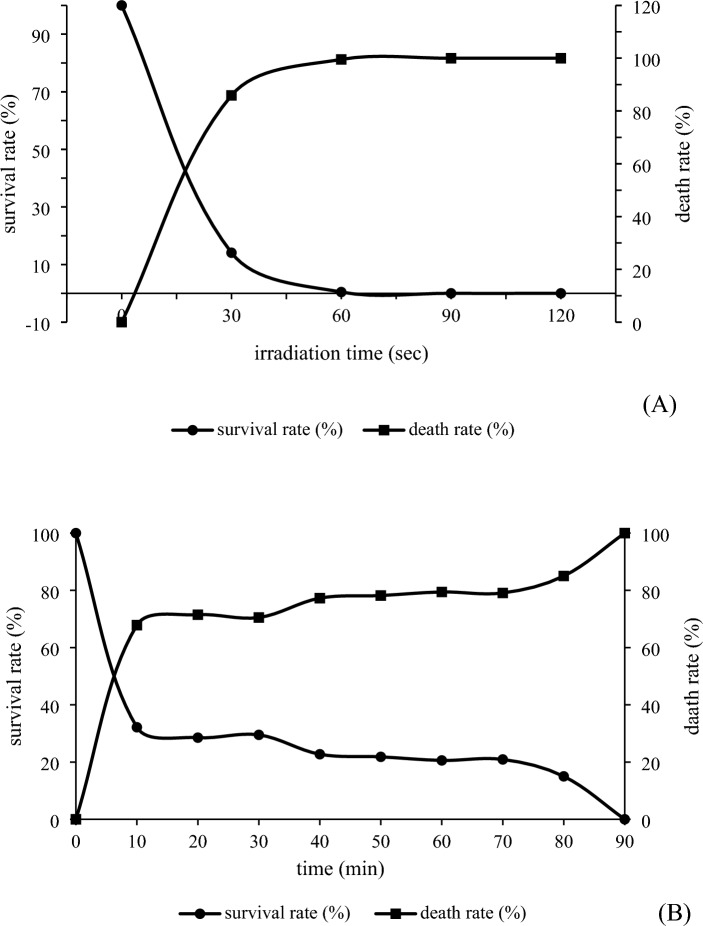
Survival rate and death rate of *W. anomalus* YWP1-3 after UV (**A**) and EMS (**B**) mutagenesis.

**Figure 2:**
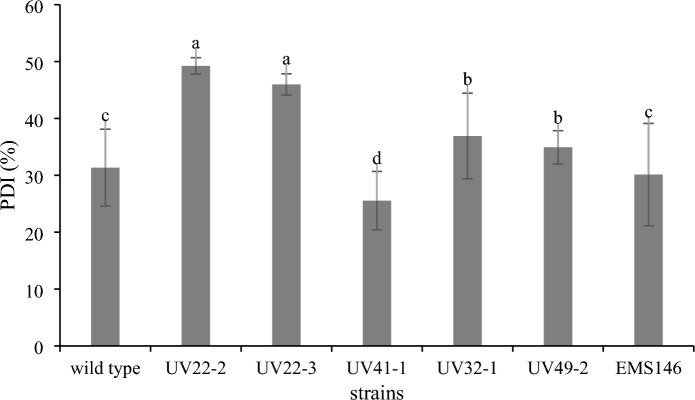
Pectin degradation index (PDI) value of *W. anomalus* YWP1-3 (wild-type) and all 6 selected mutants and wild-type on pectin agar after incubation for 4 days. Different letters (a–c) indicate statistical differences among different samples (*p* < 0.05).

The wild-type strain of *W. anomalus* YWP1-3 was subjected to UV mutagenesis by irradiation and subsequently analyzed by a pectinase production assay. The results revealed a significant decrease in the number of surviving yeast colonies with increasing UV exposure time (Fig. 1A). The data indicated that only 10% of the cells survived after 30 s of UV irradiation, and this survival rate further decreased to 3% after 60 s. Notably, at an exposure time of 120 s, the survival rate decreased to 0.02%. Further investigation of *W. anomalus* YWP1-3 was conducted using EMS mutagenesis. The results revealed a correlation between the incubation time of YWP1-3 cells with EMS and the percentage of death, as depicted in Fig. 1B. As the incubation time increased from 0 to 90 min, the survival rate steadily decreased from 100 to 0%. Based on these findings, an incubation time of 60 min with EMS was chosen as the optimal condition for screening mutant yeast strains. A total of 38 strains exhibiting pectinase activity on pectin agar were identified during the screening process. A total of 205 mutant strains, comprising 167 strains from the UV mutation group and 38 strains from the EMS mutation group, were subjected to screening based on their ability to produce pectinolytic enzymes. The screening process involved the replica plating technique and Gram's iodine assay on pectin agar. Among the mutants, six strains (UV22-2, UV22-3, UV41-1, UV32-1, UV49-2, and EMS146) exhibited relatively high pectinase degradation indices (PDIs). Notably, five mutants were derived from UV mutagenesis, while only one mutant was obtained from EMS mutagenesis. The UV22-2 and UV22-3 mutants displayed the highest PDI values of 49.22% and 45.97%, respectively, as shown in Fig. 2. These six mutants were selected for further characterization to assess their suitability as starter cultures for fermentation of Arabica coffee.

### Characterization of *W. anomalus* YWP1-3 mutant strains

#### Morphological study of mutant strains

The wild-type and six selected mutant strains were examined and evaluated for various characteristics, including size, color, texture, elevation, form, and margin, as shown in Table 1. UV22-2, UV22-3, and UV32-1 had moderate colony sizes, were cream colored, and had smooth textures and surfaces. UV41-1, UV49-2, and EMS146 had rough colony surfaces. Additionally, light microscopy was used to study the morphological features of the yeast cells. All the mutant and wild-type strains exhibited oval yeast cell shapes and budding reproduction.

**Table 1:** Morphology of *W. anomalus* YWP1-3 (wild type) and mutants cultured on YMA agar for 48 h.

Characteristics	Yeast strains						
	Wild type	UV22-2	UV22-3	UV41-1	UV32-1	UV49-2	EMS146
Colony size	Moderate	Moderate	Moderate	Moderate	Moderate	Moderate	Moderate
Color	Cream	Cream	Cream	Cream	Cream	Cream	Cream
Texture	Smooth	Smooth	Smooth	Smooth	Smooth	Smooth	Smooth
Elevation	Convex	Convex	Convex	Convex	Convex	Convex	Convex
Form	Round	Round	Round	Round	Round	Round	Round
Margin	Entire	Entire	Entire	Entire	Entire	Entire	Entire
Surface	Rough	Smooth	Smooth	Rough	Smooth	Rough	Rough

#### Growth evaluation of mutant strains in various culture media

**Figure 3:**
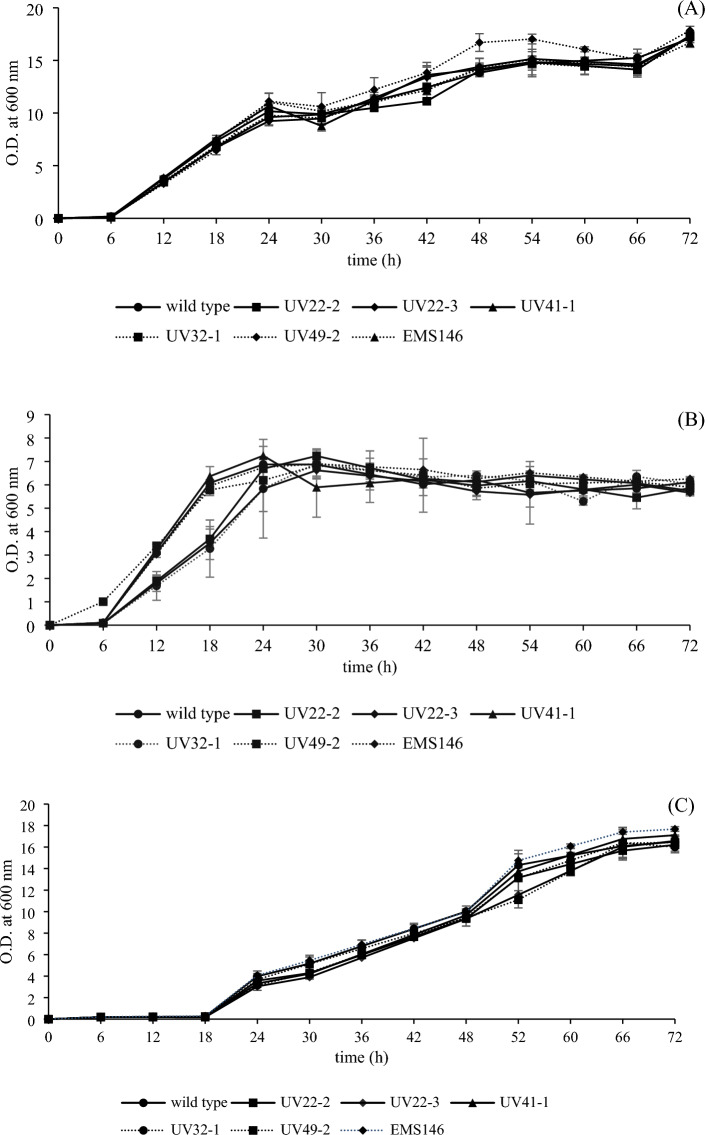
Growth curves of *W. anomalus* YWP1-3 (wild type) and 6 mutant strains in YM broth (**A**), pectin broth (**B**), mucilage broth (**C**) after incubation at 30 °C for 72 h.

The growth of both the wild-type strain and selected mutant strains was assessed in various culture media. Overall, most of the mutants and the wild type displayed similar growth patterns, consisting of lag, log, stationary, and death phases. However, in YM broth, mutant UV 49–2 exhibited significantly greater growth than the other mutants and the wild type between 48 and 54 h of cultivation (Fig. 3A). Conversely, in pectin broth, UV22-2, UV22-3, and UV32-1 showed significantly lower growth than the other mutants and the wild type after incubation for 6–24 h (Fig. 3B).

#### Enzyme production of mutant strains in mucilage broth

**Figure 4:**
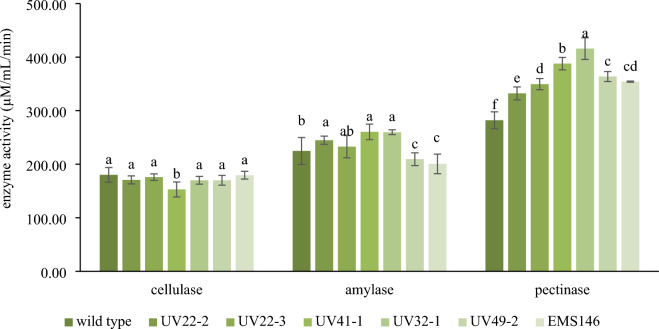
Cellulase, amylase and pectinase production in mucilage broth of *W. anomalus* YWP1-3 (wild type) and mutants after incubation at 30 °C for 72 h. Different letters (a–d) indicate statistical differences among different samples in each group; cellulase, amylase and pectinase (*p* < 0.05).

Cellulase, amylase, and pectinase production in mucilage broth was evaluated for all selected mutants and the wild type. As shown in Fig. 4, there were no significant differences in cellulase activity between the mutants and the wild type. However, strain UV41-1 showed the lowest cellulase activity. Conversely, mutants UV22-2, UV22-3, UV41-1, and UV32-1 exhibited higher amylase activities than the wild type, indicating that mutagenesis may have influenced the genes related to amylase production. Notably, all the mutants displayed substantial pectinase activity, ranging from 332.35 to 415.88 U/ml. Among these mutants, UV32-1 had the highest pectinase activity in the mucilage broth, reaching 415.88 U/ml.

#### Sugar assimilation ability of mutant strains

**Figure 5:**
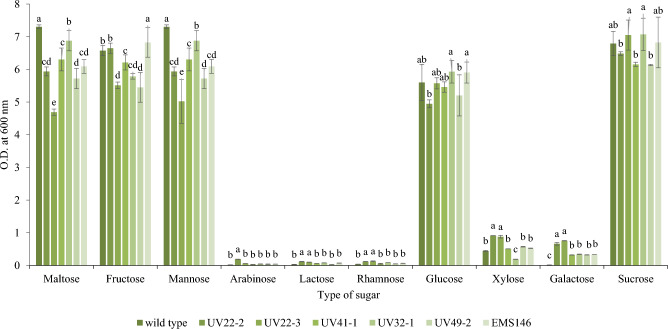
Growth of *W. anomalus* YWP1-3 (wild type) and mutants in nitrogen yeast base medium containing different sugars after incubation at 30 °C for 24 h. Different letters (a–g) indicate statistical differences among different samples in each group (*p* < 0.05).

The ability to efficiently utilize various sugars is an important characteristic when screening yeast strains for coffee fermentation. As depicted in Fig. 5, both the wild type and the mutants demonstrated the capacity to utilize all 10 sugars, as evidenced by the high optical density at 600 nm. However, variations in sugar utilization capabilities were observed between the mutants and the wild type. Specifically, compared with the wild type, the UV22-2 mutant exhibited significantly greater assimilation of arabinose. Additionally, the growth rates of the mutants UV22-2 and UV22-3 were significantly greater than those of the wild type in nitrogen yeast base media supplemented with lactose, rhamnose, and xylose. Moreover, all the mutants demonstrated significantly greater galactose utilization than did the wild type.

#### Evaluation of mutant strains as the starter for improving Arabica coffee flavor quality

This study highlights the development of a wet fermentation process utilizing starter cultures derived from UV-induced mutations. Based on its ability to produce pectinase on plates, amylase in mucilage broth, and significant growth in various sugars, UV22-2 and UV22-3 were selected and used as starters for Arabica coffee fermentation. Fragrance/aroma, flavor, acidity, body, uniformity, balance, sweetness, clean cup, and overall impression were evaluated by certified Q-Arabica graders. In the case of inoculated fermentations with UV22-1 and UV22-3, Arabica coffee beverages exhibited a pronounced cupping score, garnering higher scores compared to the wild type (80.25 ± 1.65), uninoculated (75.5 ± 2.5), and commercial coffee (72.5 ± 2.0) conditions. The cupping scores of coffee derived from UV22-2 and UV22-3 were 83.5 ± 1.5 and 82.0 ± 2.14, respectively.

#### Analysis of volatile compounds in roasted coffee by gas chromatography–mass spectrometry (GC–MS)

The volatile compound profiles of three samples of fully washed roasted coffees (wild type, UV22-2, and UV22-3) compared with those of the control and commercially available roasted coffees were analyzed using headspace solid-phase microextraction (HS-SPME) in combination with gas chromatography–mass spectrometry (GC‒MS). The GC‒MS chromatograms and volatile compound identification results are shown in Table 2. The results showed that the volatile compound profiles and identification of all the samples were similar. The most abundant compounds in the volatile profiles of all 3 samples were furan compounds (47.52–51.85%), followed by pyrazines (22.32–27.98%), ketones (3.03–4.06%), pyrroles (1.27–2.74%) and aldehydes (0.34–1.00%). Similarly, among the volatile compounds detected in the control roasted coffee, furans were present at the highest level (50.95%), followed by pyrazines (25.64%), ketones (3.33%), pyrroles (1.47%) and aldehydes (1.00%). Similarly, commercially roasted coffee also contains furans (47.52%), a major class of volatile compounds, followed by pyrazines (23.75%), ketones (3.03%), pyrroles (2.74%) and aldehydes (0.34%). Furans represented the main group of volatile compounds. The total % area of furans in sample UV22-2 (51.85%) was greater than that in the control (50.95%) and commercial (47.52%) roasted coffees. The most abundant furan compound in UV22-2 was furfuryl alcohol (16.56%), followed by furfural (11.64%) and furfuryl acetate (8.79%).

**Table 2:** Relative percentages of peak area of different chemical groups of volatile compounds in roasted coffees.

RT	Compounds	Relative percentages of peak area (% area)				
		Commercial	Control (without inoculum)	Fully washed process roasted coffee sample		
				Wild type	UV22-2	UV22-3
	**Ketones**					
1.9571	2,3-Butanedione		0.4116	0.3612	0.3442	0.3438
12.0931	2-Butanone, 1-(acetyloxy)-	1.0781	1.2637	1.7	1.2241	1.3887
2.9321	2,3-Pentanedione		1.2658	1.3172	1.158	1.1244
3.1628	2-Butanone, 3-hydroxy-		0.3931	0.5	0.41	0.42
17.7436	Ethanone, 1-(1-cyclohexen-1-yl)-	1.9527				
22.1848	4-(4'-Fluorophenyl)-3-butyn-2-one			0.1857		
	**Total**	3.0308	3.3342	4.0641	3.1363	3.2769
	**Furans**					
2.0494	Furan, 2-methyl-(furfuryl alcohol)	1.1252	0.7468	0.7958	0.7124	0.5933
6.3994	furfural	2.5824	11.8999	11.6369	11.6403	11.1006
7.6608	2-Furanmethanol	18.606	15.7443	16.6515	16.5628	15.9682
9.214	2-FURYLMETHYL FORMATE	1.3969	0.951	0.8986	1.082	0.9224
11.3224	1-(2-Furyl)-2-propanone	0.7096	0.4288	0.4077	0.4881	0.4212
11.8849	2-Furancarboxaldehyde, 5-methyl-	3.8747	9.622	8.2485	9.6145	9.7792
13.3951	2-Furanmethanol, acetate (Furfuryl acetate)	15.4963	8.6445	6.9459	8.7904	7.4602
17.4674	Furan, 2,2′-methylenebis-	1.423	0.6314			0.61
18.5885	2,5-Furandione, 3-ethyl-4-methyl-	0.4019				
22.3053	2,3-Dihydro-6-methylthieno[2,3c]furan				0.1843	
22.3073	2,3-Dihydro-6-methylthieno[2,3c]furan					0.2066
27.6944	Furan, 2,2′-[oxybis(methylene)]bis-	0.1953				
5.4042	3(2H)-Furanone, dihydro-2-methyl-	1.155	1.483	2.3075	1.9803	1.3436
11.0278	2,5-Dimethyl-3(2H)furanone	0.5531	0.8032	0.8626	0.7982	0.9278
	**Total**	47.5194	50.9549	48.755	51.8533	49.3331
	**Pyrazines**					
3.6348	Pyrazine (CAS)	0.295	0.3849		0.3331	0.3614
5.9127	2-methyl pyrazine	4.9234	6.7311	6.4241	6.2458	6.85
9.454	Pyrazine, 2,5-dimethyl-	9.83	14.4	11.1018	13.22	15.52
9.5083	Pyrazine, ethyl-	2.9168				
9.6735	Pyrazine, 2,3-dimethyl-	2.2699	1.3495	1.4409	1.3531	1.5042
13.4611	Pyrazine, 2-ethyl-5-methyl-			0.8934		0.6716
13.5639	Pyrazine, 2-ethyl-3-methyl-			0.7266		
13.5287	Pyrazine, trimethyl-	2.3121	1.9868	1.248	1.8228	2.2249
17.2186	Pyrazine, 3-ethyl-2,5-dimethyl-	1.2015	0.7852	0.4897	0.6911	0.851
	**Total**	23.7487	25.6375	22.3245	23.6659	27.9831
	**Phenols**					
11.5203	Phenol, 2,3-dimethyl- (2,3-Xylenol)					
26.6236	Guaiacol, 4-ethyl-	0.2527				
28.2047	2-Methoxy-4-vinylphenol (4-vinyl guaiacol)	0.3789				
	**Total**	0.6316				
	**Pyrroles**					
3.7533	1H-Pyrrole, 1-methyl-	0.2296	0.2256		0.1899	
13.6503	1H-Pyrrole-2-carboxaldehyde, 1-methyl-	1.5015	0.9545	1.1357	1.0434	1.0393
16.772	Ethanone, 1-(1H-pyrrol-2-yl)-	0.49				
16.967	Ethanone,1-(1-methyl-1H-pyrrole-2-yl)-	0.52				
22.1767	1H-Pyrrole, 1-(2-furanylmethyl)-		0.2861	0.19	0.3251	0.2333
	**Total**	2.7411	1.4662	1.3257	1.5584	1.2726
	**Aldehydes**					
2.4501	Butanal, 3-methyl-	0.3448	0.5455	0.5142	0.4224	0.5205
15.4811	Benzeneacetaldehyde		0.4559		0.4672	0.4429
	**Total**	0.3448	1.0014	0.5142	0.8896	0.9634
	**Others**					
2.3781	Acetic acid	3.0866	5.0537	5.0591	5.0476	4.7251
3.891	Pyridine	4.0877	0.764	0.9382	1.0252	0.9357
18.894	2-Indanone, hexahydro, trans-	0.26				
12.9703	1,6-Octadiene, 7-methyl-3-methylene				0.2625	
	Beta-myrcene	0.35		0.2487		
22.3061	3-Ethyl-2-formylthiophene	0.17	0.1859	0.18		
	**Total**	7.9543	6.0036	6.426	6.3353	5.6608

## Discussion

This work revealed UV and EMS mutants of *W. anomalus* YWP1-3. As the exposure time to UV radiation increased, there was a significant decrease in the number of surviving colonies of *W. anomalus* HH16, as indicated by a recent report on UV mutation results. Moreover, only 12% of the yeast colonies survived after 5 min of UV irradiation, 5% survived after 10 min, and only one yeast colony survived after 15 min, whereas at a UV exposure time of 20 min, no colonies were reported^18^. In our work, 167 mutants from UV exposure were detected. Five mutants were selected based on their ability to produce pectinase activity on a pectin agar plate. Hawary et al.^18^ reported that 2 surviving mutants were selected for glycerol production based on resistance to exogenous ethanol, in contrast to the wild-type isolate in media supplemented with 10–30% (v/v) ethanol. The higher pectinase production on the pectin agar of the selected mutants than of the wild type might be due to yeast mutation during UV irradiation^19^. Induced UV mutation is the most straightforward and highly efficient physical method for identifying genetic mutations. These mutations, alterations in the genetic code, represent a vital wellspring of diversity within the context of evolutionary processes. Ethyl methanesulfonate (EMS), an organic compound known to be mutagenic, teratogenic, and carcinogenic, plays a central role in this process. EMS induces random mutations in genetic material through nucleotide substitution, with its primary outcome being point mutations. Owing to its remarkable potency and well-understood spectrum of mutational effects, EMS is the most widely utilized chemical mutagen in experimental genetics. Mutations triggered by exposure to EMS can subsequently be detected through genetic screens or other relevant assays^20^. In the context of this study, the utilization of UV and EMS mutation techniques is favored because they do not fall under the classification of genetically modified microorganisms (GMMs). Both of these mutation methods offer precise control and can be manipulated conveniently on a laboratory scale without posing any harm to the operator. The literature contains a wealth of reports showing the success of UV and EMS mutations in enhancing the production of industrial goods. However, it is worth noting that no reports to date have explored the mutation of starter cultures for the fermentation of Arabica coffee. Nonetheless, in 2019, UV irradiation was employed to augment glycerol production in *Wickerhamomyces anomalus* HH16^18^. In a separate study by Revin et al.^19^, a notable increase in the saccharification of starchy raw materials and the fermentation of wort into ethanol was achieved through a two-stage mechanical grinding process and ultraviolet pretreatment of yeast. UV irradiation has the capacity to induce mutagenic and cytotoxic DNA lesions, including cyclobutane–pyrimidine dimers (CPDs) and 6–4 photoproducts (6–4 PPs). However, yeast cells have evolved an extensive array of DNA damage repair mechanisms to counteract the deleterious effects of UV exposure. Among these mechanisms, photoreactivation involving the photolyase enzyme is recognized as one of the most effective repair strategies developed by yeasts^21^. The concept of random mutagenesis in yeast, facilitated by mutagenic agents and UV light, holds significant promise for enhancing various processes, including lipid production^22^, ethanol production^23^, and sugar alcohol production^24^.

The pectinolytic activity on pectin agar was evaluated and used for the screening of mutant strains in this study. The mucilage layer covering depulped coffee beans comprises various components, with 84.2% water, 8.9% protein, 4.1% sugar, 0.91% pectic substances, and 0.7% ash^25^. Further analysis of its polysaccharide composition revealed that the alcohol-insoluble fraction consisted of 30% pectin, 8% cellulose, and 18% neutral noncellulosic polysaccharides and was composed of monosaccharides such as arabinose, xylose, galactose, and other simple sugars^7^. Essential aroma precursors, including sugars, proteins, amino acids, and phenolic compounds, are naturally present in green coffee beans and contribute significantly to the formation of the coffee aroma. During the wet fermentation process, microorganisms breakdown mucilage, yielding extracellular mucilage-degrading enzymes such as pectinase, protease, and cellulase^26^. The key enzymes involved in coffee fermentation are polygalacturonase (PG), pectin lyase (PL), and pectin methylesterase (PME)^27^. These enzymes have the potential to fully digest pectin, producing galacturonic acid and its oligomers^28–30^. Recently, researchers^10,31^ successfully identified a potential starter culture for coffee fermentation based on criteria such as pectinase production, growth in mucilage broth, and sugar assimilation. Strain YWP1-3 exhibited pectinase activity and received high scores in the Arabica coffee sensorial tests. In a separate study, Haile and Kang^27^ reported on *Wickerhamomyces anomalus* KNU18Y3, which was isolated from a wet fermentation process and displayed promising capabilities for the production of pectinase enzymes, including polygalacturonase and pectin lyase.

The morphology of the selected mutants was studied and was shown to be similar to that described in a previous report. Aerobic cultivation resulted in colonies of *W. anomalus* that exhibit a color spectrum from white to tannish-white, typically displaying a butyrous texture. Variations in strains are observed, with some appearing smooth and glistening, while others exhibit a dull and somewhat chalky appearance. Colony margins vary from entirely smooth to lobed, occasionally featuring a fringe of pseudohyphae. In addition, these cultures typically emit a faintly pleasant odor^32^.

All the mutants and the wild type displayed similar growth characteristics in mucilage broth. Krajangsang et al.^10^ effectively screened isolated yeast strains to identify a suitable starter culture for the fermentation of Arabica coffee. They assessed yeast growth by evaluating its performance on pectin agar, mucilage broth, and other relevant characteristics. This implies that assessing growth under different conditions can aid in the identification of potential strains for coffee fermentation.

Most of the mutant strains showed no significant differences in cellulase activity when compared to that of the wild type. The mucilage composition, which consists of a small amount of cellulose, did not significantly affect cellulase production in the mucilage broth of any of the strains^7^. Cellulase is an inducible enzyme responsible for breaking down cellulose molecules into monosaccharides, such as β-glucose, as well as shorter polysaccharides and oligosaccharides^33^. UV32-1 had the highest pectinase activity in the mucilage broth, reaching 415.88 U/ml. This finding suggested that mutagenesis effectively bolstered pectinase production in this specific mutant. In a related context, Liu et al.^34^ reported that mutant JU-A10-T exhibited a ninefold increase in cellulase activity (measured as filter paper enzyme, FPase), an eightfold increase in xylanase activity (a major type of hemicellulase), and a fourfold increase in total secreted proteins when compared to the wild-type strain 114–2 in cellulose-wheat bran (CW) medium, a standard medium for cellulase production in *P. decumbens*. However, the amount of β-glucosidase produced by the mutant strain was lower than that produced by the wild type, similar to our findings with strain UV41-1. Using submerged fermentation techniques, Antier et al.^35^ demonstrated that the dgrAW99-iii mutant produced 300% more pectinase than did the wild type strain *Aspergillus niger* C28B25. Lima et al.^36^ showed the enhanced pectin degradation abilities of *Penicillium griseoroseum* mutants (M02, M03, M04, M05, and M07) and selected sectors (M03 and M05) for pectin lyase (PL) production in liquid media due to their larger pectin degradation zones compared to those of the wild type.

The mucilage composition, which consists of a slight amount of cellulose affecting cellulase production in the mucilage broth of all strains, was not significantly different between strains^7^. Cellulase is an inducible enzyme responsible for breaking down cellulose molecules into monosaccharides, such as β-glucose, as well as shorter polysaccharides and oligosaccharides^33^. These findings suggest that neither UV nor EMS mutagenesis significantly influenced the genes associated with cellulase production. It is conceivable that the mutations primarily affected noncoding regions or had no effect on the nucleotides responsible for cellulase production. Synonymous and stop-gain mutations in the mutants might result in comparable levels of enzyme production.

Moreover, all the mutants demonstrated significantly greater galactose utilization than did the wild type. These findings indicate that the mutagenesis process influenced the sugar assimilation abilities of the mutants, potentially rendering them more suitable for coffee fermentation. The sugar compounds found in the coffee mucilage were maltose, fructose, mannose, arabinose, lactose, rhamnose, glucose, xylose, galactose, and sucrose. Arabica mucilage was analyzed and showed abundant sugar consisting of 35.65 g/L glucose, 36.67 g/L galactose and 1.06 g/L lactose^37^. Arabica mucilage collected in Mexico was shown to contain 9.6% rhamnose, 1.3% fructose, 52.5% arabinose, 8.9% xylose, 0.8% mannose, 19.7% galactose and 7.8% glucose^6^. The sugar assimilation efficiency of mutants is one of the characteristics used to screen a potential strain for coffee fermentation. Krajangsang et al.^10^ reported that the sugar utilization ability of selected yeast for coffee fermentation was an important factor for screening potential strains for coffee fermentation.

The cupping scores of coffee derived from UV22-2 and UV22-3 were 83.5 ± 1.5 and 82.0 ± 2.14, respectively. This outcome suggested that the mutant strains contributed distinct and favorable flavors to the coffee products. The cupping notes indicated that coffee fermented with UV22-2 and UV22-3 exhibited elevated sweetness, moderate acidity, substantial body weight, a lingering aftertaste, and floral and fruity flavors. This could be attributed to the robust pectinase production of UV22-2 and UV22-3, which can release more sugar from coffee beans. Additionally, pectinase is prevalent in mucilage broth, and its superior carbon utilization ability results in good flavor quality in the final cup^38^. Various reports have successfully improved the flavor quality of Arabica coffee by using starters such as *Wickerhamomyces anomalus* KNU18Y3 and *Wickerhamomyces anomalus* YWP1-3^9,10^. Moreover, yeast starters such as *Saccharomyces cerevisiae* and *Pichia kudriavzevii* were applied for Robusta coffee (*Coffea robusta*) fermentation under controlled fermentation in a bioreactor^39^.

The flavor profile of fermented coffee was evaluated by GC–MS. The characteristic flavors of furfuryl alcohol and furfuryl acetate are known as sweet, fruity, caramel-like, and roasted flavors^38,40^. These furan derivatives could significantly contribute to increasing sweet, fruity, and caramel-like aromas in sample UV22-2. Pyrazines, the second majority of volatile compounds, were found in all coffee samples. Alkylpyrazines contributed to the nutty and roasted aromas. Compared with the control and commercial coffees, UV22-3 coffee contained the lowest odor threshold compounds 2-methylpyrazine and 2,5-dimethylpyrazine in the highest amounts, which contributes to cocoa, nutty, and roasted aromas^41^. Ketones, which lend buttery and caramel-like flavors, were detected in all the roasted coffee in this study^42^. The wild-type sample contained the highest content of ketones (4.06%), such as 1-acetyloxy-2-butanone and 2,3-butanedione, which contribute to more buttery and caramel-like aromas than the other samples^43^. Other minor volatile compounds identified in all the samples were pyrroles, aldehydes, acetic acid, and pyridine. Because all the samples contained these compounds in small amounts, these compounds may not significantly impact their aroma. Remarkably, the roasted coffee produced using UV22-2 and UV22-3 mutant strains as starter cultures for the fermentation process exhibited higher concentrations of furans, pyrazines, pyrroles, and aldehydes than did the wild type. This suggests that the mutant strains have the potential to enhance the flavor profile of fermented Arabica coffee.

## Conclusion

In our research, we successfully manipulated a yeast strain, *Wickerhamomyces anomalus* YWP1-3, through UV irradiation and EMS treatment. Remarkably, we identified six mutant strains that exhibited distinctive characteristics compared to the wild-type strain. Notably, one of these mutants, UV22-2, emerged as a highly promising candidate for use as a starter for the fermentation of Arabica coffee. UV22-2 demonstrated the ability to produce pectinase enzymes when cultured on pectin agar medium, achieving a pectin degradation index (PDI) of up to 49.2% and an enzyme activity of 332.35 U/ml. Furthermore, UV22-2 exhibited versatility in utilizing various types of sugars as a carbon source. In the sensory cupping evaluations, coffee fermented with UV22-2 had the highest score. Additionally, the volatile compounds present in coffee roasted following fermentation by UV22-2 were analyzed using GC‒MS, revealing significantly higher levels of furfuryl alcohol and furfuryl acetate than in other samples. Consequently, our study represents a groundbreaking advancement in the development of a mutant yeast strain as an inoculant for the wet processing of coffee, ultimately leading to an enhancement in the overall flavor and quality of the resulting coffee.

## Data Availability

All data produced or examined in the course of this study have been incorporated within this published article and adhere to established research protocols.
